# Identification and Expression Analysis of the Goji Haploid-Inducible Gene DMP

**DOI:** 10.3390/ijms27062912

**Published:** 2026-03-23

**Authors:** Zijun Yang, Cuiping Wang, Zhonghua Wang, Jiali Wu

**Affiliations:** School of Biological Science and Engineering, North Minzu University, Yinchuan 750021, China; 18388860713@163.com (Z.Y.);

**Keywords:** *Lycium barbarum*, DMP gene, gene cloning, subcellular localization, expression analysis

## Abstract

Goji, a plant unique to China, is recognized for its dual use as both a food and a medicine and is rich in various nutrients. However, long-term asexual propagation often leads to cultivar degeneration and viral accumulation, which severely impact its yield, quality, and disease resistance. Homozygous seeds can stably produce offspring with uniform traits. Haploid breeding technology, which involves doubling the chromosomes of haploid plants to obtain homozygous diploids, can significantly accelerate the breeding process. The *DMP* (Domain of Unknown Function 679 Membrane Protein) family is a plant-specific family of membrane proteins involved in various biological functions, including physiological processes, reproductive development, and senescence. Concurrently, loss-of-function of the *DMP* gene impedes the proper integration of the paternal genome following fertilization. Consequently, the embryo develops with exclusively maternal chromosomes, a mechanism that underlies the induction of haploids. In this study, we conducted a genome-wide identification of the *DMP* gene family in goji, analyzing the physicochemical properties, chromosomal locations, cis-acting elements, phylogenetic relationships, sequence characteristics, expression patterns, and subcellular localization of its members. The objective was to identify *DMP* genes capable of inducing haploid production in goji berry for future breeding applications. The results revealed a total of 11 DMP family members in the goji berry genome, distributed across seven chromosomes. The proteins encoded by these members contain 136 to 237 amino acids, with molecular weights ranging from 15,267.96 to 26,141.01 Da and isoelectric points (pI) ranging from 5.14 to 9.32. The LbDMPs were found to contain numerous cis-acting elements that play roles in plant responses to abiotic stresses and various phytohormones. Notably, LbDMP1 and LbDMP11, which contain the typical DUF679 domain, are predominantly expressed in pollen, suggesting their involvement in the reproductive process of goji berry. They were therefore identified as candidate genes for haploid induction. Subcellular localization analysis demonstrated that LbDMP1 is localized to the plasma membrane, while LbDMP11 is localized to membrane systems such as the endoplasmic reticulum. This research provides a fundamental basis for further exploration of the functional roles of the *DMP* gene family in goji berry and offers valuable genetic resources for haploid induction in its breeding programs.

## 1. Introduction

Goji, a deciduous shrub belonging to the genus *Lycium* L. in the *Solanaceae* family, is a plant of significant importance for both medicinal and culinary purposes. It is recognized as one of the most economically and medicinally valuable fruit crops in the world [1,2,3,4]. The genus *Lycium* L. comprises over 80 species globally, with China being home to seven species and three varieties, primarily distributed in the northwestern regions, including Ningxia, Qinghai, Gansu, and Xinjiang [5]. Goji is rich in various nutrients, such as *Lycium barbarum* polysaccharides, carotenoids, and flavonoids, and possesses multiple health-promoting functions, including nourishing the liver and kidneys, enhancing immunity, and regulating blood glucose and lipid levels [6]. As of 2023, the cultivation area of goji in China reached 1.83 million mu (approximately 122,000 hectares), with an annual fresh fruit yield of about 1.4 million tons, a dried fruit yield of approximately 240,000 tons, and a total output value exceeding 29 billion RMB. However, long-term asexual propagation has led to a reduction in the adaptability of elite goji varieties, a decline in stress resistance, and the accumulation of deleterious mutations, which severely impact their yield, quality, and overall utility. Unsuitable cultivation conditions significantly affect the growth of goji, leading to the loss of superior characteristics. However, it remains unclear how these factors affect vegetatively propagated progeny. Furthermore, as a cross-pollinated plant, goji shows frequent natural pollination. This results in significant trait segregation in seed progeny due to genetic recombination, making it difficult to rapidly obtain pure lines. Additionally, conventional breeding requires approximately 10 years, restricting variety renewal. In contrast, haploid breeding enables rapid homozygosity, shortening the breeding cycle. Simultaneously, haploid cells may undergo natural virus elimination, reducing the risk of vertical transmission.

Membrane proteins are key executors in regulating fundamental life processes, including cell proliferation, signal transduction, and material transport. The DMP (Domain of Unknown Function 679 membrane proteins) family, a plant-specific gene family encoding membrane proteins, has increasingly become a research focus [7,8,9,10]. Members of this family are deeply involved in multiple physiological processes, such as plant reproductive development and senescence regulation [11,12]. In the model plant *Arabidopsis thaliana*, for instance, the functions of its 10 DMP members are clearly differentiated: AtDMP1, AtDMP2, AtDMP3, AtDMP4, and AtDMP7 primarily mediate programmed cell death, whereas AtDMP8 and AtDMP9 specifically promote gamete fusion during fertilization [13,14]. This functional diversity is also corroborated in other species. For example, 33 *AsDMP* genes in *Medicago sativa* have been confirmed to be associated with seed aging [15], while DMPs in cotton are predicted to play multiple roles in senescence, reproduction, and stress responses [16]. The most remarkable application potential of *DMP* genes lies in their ability to induce haploids. The core mechanism is that the loss of *DMP* gene function (e.g., through gene knockout or mutation) disrupts the normal developmental trajectory of plant gametes (pollen or egg cells). This disruption triggers parthenogenesis or androgenesis, ultimately leading to the production of plants with a single set of chromosomes. This breakthrough has been successfully validated in several important crops, including *Arabidopsis thaliana*, *Solanum lycopersicum*, *Solanum tuberosum*, *Zea mays*, *Brassica napus*, and *Nicotiana tabacum*, thereby establishing efficient maternal haploid induction systems for these crops [17,18,19,20,21,22].

Haploid breeding offers a promising solution to address the issues of virus accumulation, mutation buildup, and cultivar degeneration that arise from the long-term asexual propagation of goji. We aim to rapidly obtain pure lines of superior cultivars through haploid breeding to accelerate the breeding process and enable seed-based propagation. The first critical step is to identify the *DMP* gene associated with haploid induction in goji. Therefore, this study aims to achieve the following specific objectives: (1) To conduct a genome-wide identification and analysis of the DMP gene family in goji. (2) To screen and identify candidate *DMP* genes that are potentially involved in the haploid induction process in goji. The results of this research will lay the foundation for in-depth functional analysis of the DMP gene family and provide essential genetic resources and a theoretical basis for the ultimate establishment of a haploid breeding system for goji.

## 2. Results

### 2.1. 11 LbDMP Genes Were Identified in the Whole Genome of Goji

The physicochemical properties of the DMP protein family in goji are presented in Table 1. The proteins encoded by the *LbDMP* genes range from 136 to 237 amino acids in length, with molecular weights ranging from 15,267.96 to 26,167.96 Da and isoelectric points (pI) from 5.14 to 9.32. With the exception of LbDMP3, LbDMP4, and LbDMP5, which have instability indices greater than 40 and are classified as unstable proteins, the remaining LbDMPs have instability indices below 40, indicating they are stable. The aliphatic index values for the proteins range from 89.21 to 108.16, and the grand average of hydropathicity (GRAVY) scores range from 0.093 to 0.587. Subcellular localization prediction indicated that all DMPs are localized to the plasma membrane, which is consistent with the known functions of DMPs in other species.

### 2.2. Chromosomal Localization an Collinearity Analysis of LbDMP Genes

**Figure 1:**
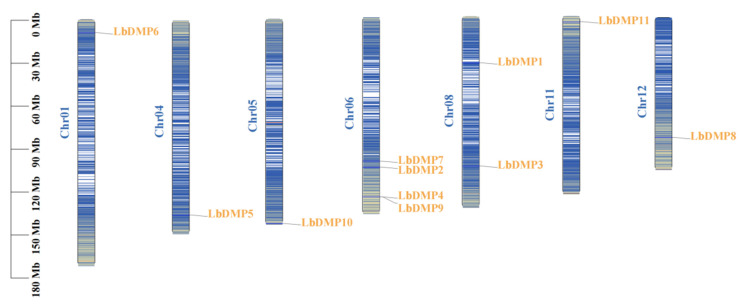
A chromosome mapping analysis of the *LbDMP* gene family. Chromosome numbers are shown in blue font. The *LbDMP* gene is shown in yellow font on the side. The scale is marked in trillion bases (Mb), the color of the chromosomes from blue to red represents gene density from small to large.

**Figure 2:**
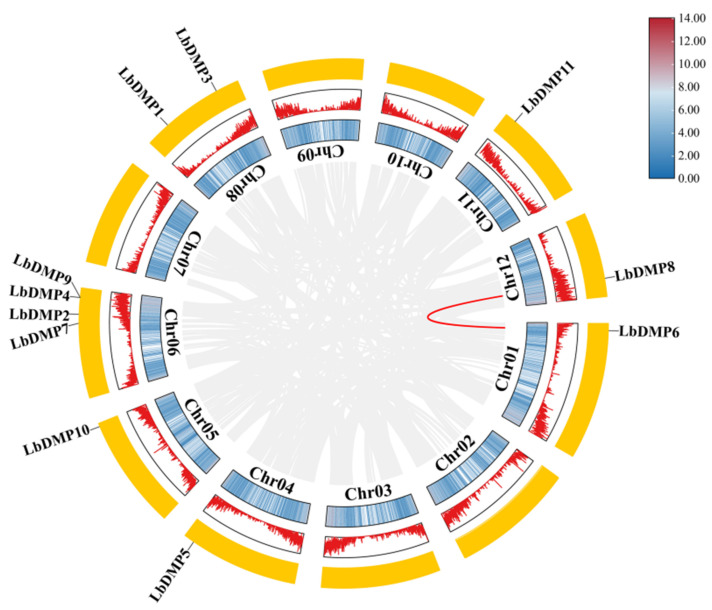
Chromosome distribution and replication events of LbDMPs. Duplicate genes are connected by red lines with reference to the 11 genes highlighted in black. The panel in the upper right corner illustrates the chromosome density across the region. The color scale denotes density levels, with red signifying the highest density and blue the lowest. In the circular genome map, all three circular layers represent chromosomes. The innermost layer is a density heatmap, the middle layer uses a peak plot to represent gene density, and the outermost yellow layer is used to mark chromosome boundaries.

**Figure 3:**
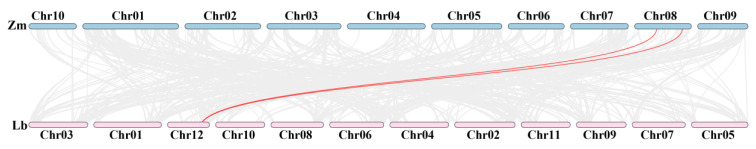
Collinearity analysis of goji and maize. Zm, *Zea mays*, Lb, *Lycium barbarum*. Red lines highlight the *DMP* gene pairs.

Based on their positional information within the goji genome, a schematic diagram of the chromosomal locations of the *LbDMP* genes was constructed (Figure 1). The results indicated that the 11 *LbDMPs* gene are unevenly distributed across seven chromosomes. Chromosome 9 contains the highest number of *LbDMP* genes (4), followed by chromosome 8 with two genes, while the remaining chromosomes each contain a single *LbDMP* gene. Notably, the majority of *LbDMP* genes exhibit a distinct tendency to be concentrated at the chromosomal termini. To elucidate the expansion mechanisms of the *LbDMP* gene family, a homology analysis was conducted to visualize the relationships among these genes (Figure 2). This analysis confirmed the uneven distribution of LbDMPs on chromosomes 1 through 12, with chromosome 9 harboring the most genes (4), and chromosome 8 harboring two. Among the 11 genes, the homologous pair LbDMP6/LbDMP8 arose from a tandem duplication event. To investigate the evolutionary relationships of *DMP* genes across species, a whole-genome collinearity analysis was performed between goji and maize (Figure 3). The results revealed that one *LbDMP* gene in goji corresponds to two gene loci in the maize genome, each associated with a *ZmDMP* gene.

### 2.3. Cis-Acting Element Analysis of the LbDMPs Gene Family

**Figure 4:**
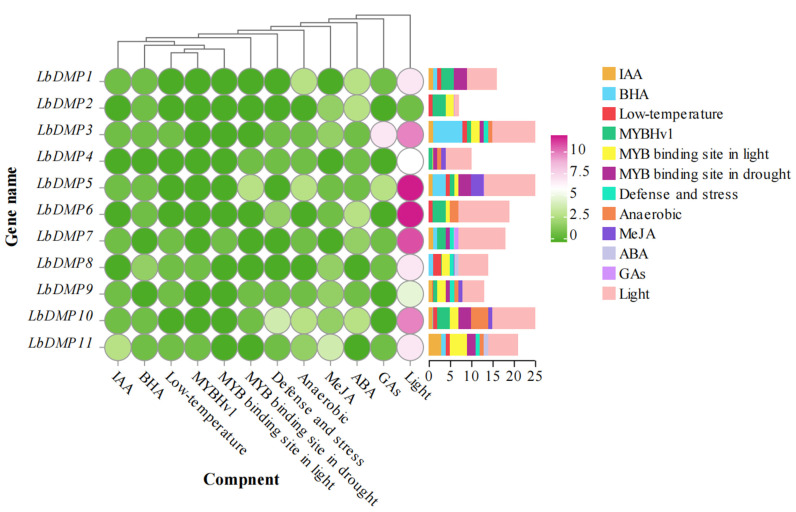
Analysis of a dynamic heatmap and stacked bar chart for cis-acting elements in the *LbDMP* gene family. In the figure, different colored blocks represent the quantity of cis-acting elements contained. Y-axis: Gene names. X-axis: Types of cis-acting elements. Circular Dot Matrix: The color intensity indicates the enrichment level of the gene for the corresponding cis-acting element. The redder the color, the greater the number of elements. Right-side Bar Chart: The colored segments represent the abundance of different cis-acting elements. The color corresponds to the element type, and the length corresponds to the abundance level of the elements contained within the gene.

Cis-acting elements, the fundamental components of promoters, dictate gene expression patterns. To investigate the regulatory mechanisms and potential functions of goji *DMP* genes, we performed a predictive analysis of cis-elements in their promoter sequences using the Plant CARE database. The identified elements were functionally categorized into three groups: hormone response, abiotic stress response, and growth and development (Figure 4). The hormone-responsive category encompassed sites for auxin, gibberellin, ABA, SA, and MeJA, while the abiotic stress-responsive category was linked to signals like low temperature, drought, anaerobiosis, and defense. Notably, the heatmap analysis revealed that light-responsive elements were the most abundant class within the growth and development-related elements. This finding indicates that the expression of goji *DMP* genes is likely under the precise control of light signals, offering key molecular evidence for their involvement in processes such as plant photomorphogenesis.

### 2.4. Phylogenetic Relationship Analysis of the LbDMP Gene Family

**Figure 5:**
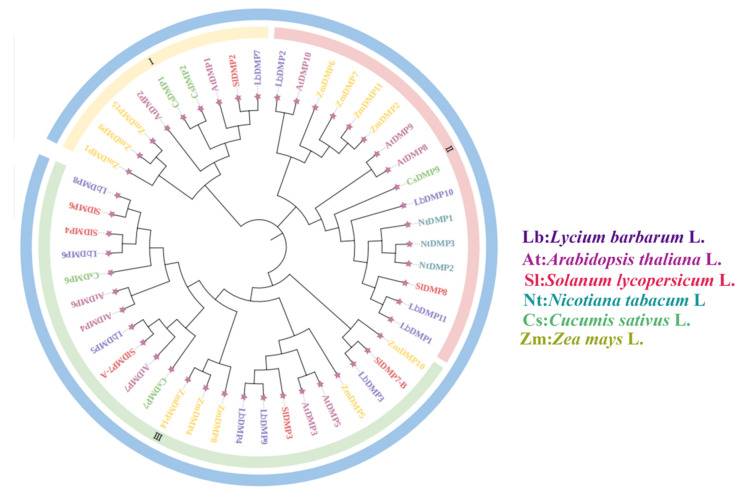
Phyloevolutionary tree analysis of the *DMP* gene family. Different colors represent different species. I, II, and III represent the three major subfamilies of the phylogenetic tree, and the pentagrams are the terminal labels for each branch.

To investigate the evolutionary relationships of the *DMP* gene family in goji, a phylogenetic tree was constructed using the *DMP* gene families from six species: Sl, Zm, At, Cs, Nt, and Lb (Figure 5). The analysis revealed that LbDMP1 (XM_060318353.1), LbDMP10 (XM_059460021.1), and LbDMP11 (XM_060333010.1) share the closest evolutionary relationship with AtDMP8 (AT1G09157.1), AtDMP9 (AT5G39650.1), SlDMP8 (Solyc05g007920.2.1), NtDMP1 (XP_016497787.1), NtDMP2 (XP_016496141.1), NtDMP3 (XP_016434048.1), and CsDMP9 (XP_004146729.1). In contrast, ZmDMP was placed on a separate clade. This distinct clustering is likely attributable to the greater evolutionary distance between maize and the other species (At, Sl, Nt, Cs, and Lb). Based on these findings, we identified LbDMP1 and LbDMP11 as our candidate genes.

### 2.5. Expression Pattern Analysis of LbDMPs

**Figure 6:**
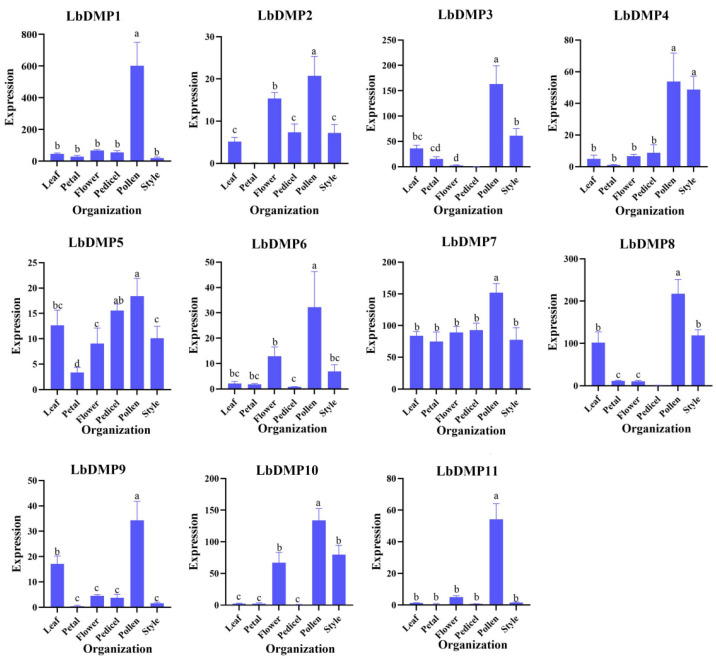
Analysis of *LbDMP* gene expression patterns. LbActin was used as the reference gene, and the relative expression of genes was calculated using the 2^−ΔΔCt^ algorithm. a, b and c in the bar graphs indicate differences in DMP expression at different sites, with identical letters indicating no significance and different letters.

To analyze the differential expression patterns of *LbDMP* genes in various organs, samples of leaves, whole flowers, petals, pedicels, pollen, and styles were collected from goji at the same developmental stage (Figure 6). The results showed that LbDMP1 and LbDMP11 were highly expressed in pollen; LbDMP2 and LbDMP6 exhibited high expression levels in whole flowers and pollen; LbDMP3 and LbDMP4 were highly expressed in pollen and styles; LbDMP5 and LbDMP7 showed no significant difference in expression across the six tissues; LbDMP8 was highly expressed in leaves, pollen, and styles; LbDMP9 was highly expressed in leaves and pollen; and LbDMP10 was highly expressed in whole flowers, pollen, and styles. The expression patterns of LbDMP1 and LbDMP11 are consistent with the specific expression profiles of their orthologs in Sl and At, which are also specifically expressed in pollen. This pollen-specific expression is considered a prerequisite for the *LbDMP* genes to function in haploid induction.

### 2.6. Cloning of LbDMPs and Analysis of Protein Conserved Domains

**Figure 7:**
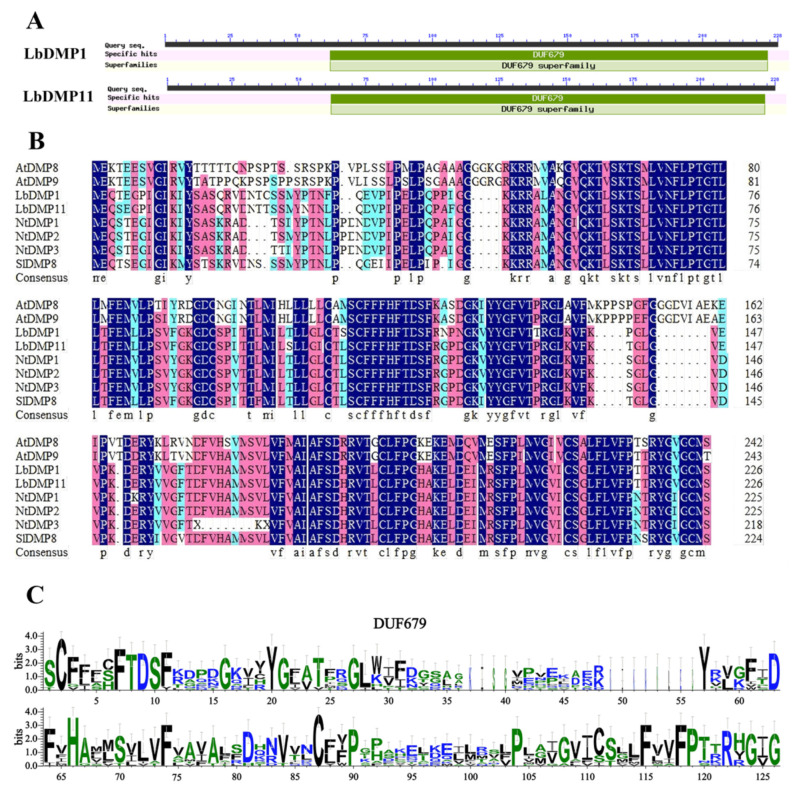
Conserved domain analysis of the DMP genes. (**A**) Conserved domain. (**B**) Multiple sequence alignment of goji, tobacco, tomato and *Arabidopsis*. (**C**) Conserved motif prediction. In (**B**), different colored blocks represent amino acids with varying degrees of similarity. In (**C**), the higher the information value, the larger the character, indicating stronger conservation at that position. Black characters represent hydrophobic amino acids, while blue and green represent hydrophilic amino acids.

The *LbDMP1* and *LbDMP11* genes were cloned to amplify their genomic DNA and coding sequence (CDS) regions. Sequencing results revealed that the CDS of LbDMP1 is 724 bp in length, while the CDS of LbDMP11 is 1084 bp. The conserved domains of the LbDMPs were analyzed using the NCBI CD-Search tool, which indicated that the *LbDMP* genes belong to the DUF679 protein family (Figure 7A). Furthermore, the amino acid sequences of AtDMP8, AtDMP9, SlDMP8, NtDMP1, NtDMP2, NtDMP3, LbDMP1, and LbDMP11 were aligned using MEGA6.06 software. The results (Figure 7B) demonstrated that the protein sequences from these four species are highly conserved within the DUF679 domain region. Finally, WebLogo 3 was used to analyze the conserved domains of LbDMPs and their counterparts from other species (Figure 7C). This analysis confirmed the presence of the DUF679 domain in all the examined species.

### 2.7. Subcellular Localization Analysis of LbDMP1 and LbDMP11 Proteins

**Figure 8:**
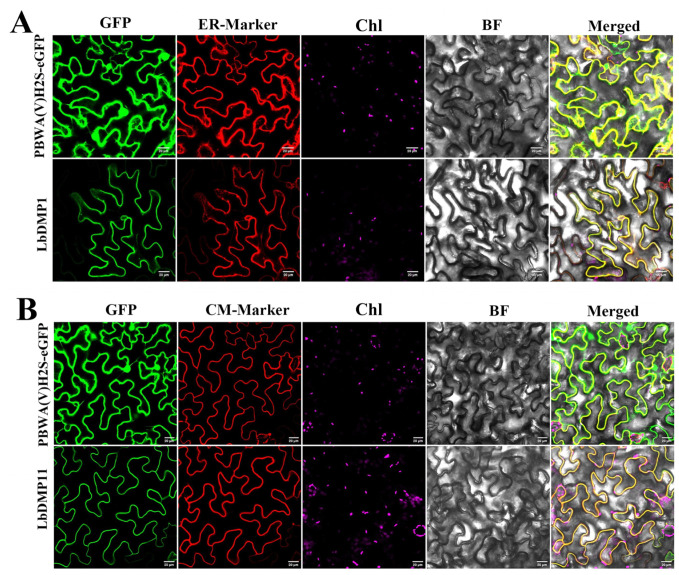
Subcellular localization of *LbDMP* genes. PBWA (V) H2S-eGFP is the blank control, and LbDMP1 and LbDMP11 are the experimental groups. The panels, from left to right, show the GFP fluorescence channel, the marker channel (ER-Marker/CM-Marker), the chloroplast channel (Chl), the bright field (BF), and the merged image. (**A**). LbDMP1 subcellular co-localization. (**B**). LbDMP11 subcellular co-localization. Scale = 20 μm.

To systematically analyze the localization of *DMP* genes, this study constructed DMP-GFP fusion expression vectors and performed subcellular localization observation via an *Agrobacterium*-mediated transient transformation system in tobacco. Confocal microscopy analysis indicated that LbDMP1 may be localized to the plasma membrane, while also showing expression in the endoplasmic reticulum (ER). In contrast, LbDMP11 was localized exclusively to the plasma membrane. To further confirm their localization, co-localization analysis was conducted. LbDMP1 was verified with an ER marker, and LbDMP11 with a plasma membrane marker. The results (Figure 8) showed that in tobacco carrying the *LbDMP1* gene, protein fluorescence signal was observed at the ER, but it did not completely overlap with the fluorescence signal of the ER marker. By comparison, in tobacco carrying the *LbDMP11* gene, the signal was most prominent in the plasma membrane region and overlapped with the fluorescence signal of the plasma membrane marker. These results indicate that both *DMP* genes are localized to broad membrane structures.

## 3. Discussion

Goji as an economic crop with significant medicinal and edible value, is rich in a variety of active ingredients. The main cultivated varieties of goji in China mostly originate from the Ningxia goji series, such as ‘Ningqi-1’ and ‘Ningqi-7’ [23]. Asexual propagation is a common method in goji cultivation. Its advantages include the ability to maintain the excellent traits of the mother plant, high propagation efficiency, and suitability for large-scale planting [24]. However, long-term asexual propagation leads to a reduction in the genetic diversity of the varieties, causing a decline in stress resistance, yield, and quality of goji. Asexual propagation materials are susceptible to virus infection. As the number of propagation generations increases, virus accumulation can lead to poor plant growth, reduced yield, and even death. Although some studies have shown that this problem can be effectively addressed in potato through meristem culture techniques and antiviral treatment protocols [25,26]. The long-term use of the same batch of asexual propagation materials may weaken the adaptability of goji to environmental changes and increase the risk of pests and diseases. Unsuitable cultivation conditions (such as high temperature and drought) also affect the growth of goji. These effects cause goji to gradually lose its original excellent traits, such as reduced yield, inferior quality, and weakened resistance. Furthermore, goji is a plant with frequent cross-pollination, and the frequency of natural pollination and fertilization between varieties is relatively high. This leads to changes in genotype frequencies, resulting in non-uniform traits in the progeny.

Haploid breeding is a modern breeding technique that involves the artificial induction of haploid plants, followed by chromosome doubling to obtain homozygous diploids. This technology can significantly shorten the breeding cycle and rapidly generate pure lines, holding significant application value in crop genetic improvement. Compared with traditional breeding methods, haploid breeding can obtain stable diploid pure lines within two generations, thus saving considerable time and cost [27]. In 2017, three research groups simultaneously published the identification of the gene MTL/ZmPLA1/NLD within the qhir1 locus of the maize haploid inducer line Stock6 [28,29,30]. Mutation of these three genes yields a haploid induction rate of ~2%. By knocking out the rice ortholog OsMTL, a rice haploid inducer line, Osmtl, was obtained [31]. Subsequently, multiple research teams edited the MTL/ZmPLA1/NLD orthologs in other monocot plants such as foxtail millet, wheat, and barley, creating corresponding haploid inducer lines for these species [32,33,34]. However, MTL/PLA1/NLD is conserved in monocot plants but not in dicot plants. In 2019, the team of Chen Shaojiang identified and cloned the *ZmDMP* gene in maize. This was the first cloned haploid induction gene, and the protein it encodes contains a DUF679 domain and is specifically expressed in pollen [20]. Building on their prior work, the research team subsequently knocked out the *Arabidopsis thaliana* orthologs, AtDMP8/9, in 2020, providing the first evidence for the functional conservation of the *ZmDMP* gene in dicot plants [17]. To further explore its potential in major dicot crops, the team identified a single ZmDMP ortholog in tomato through sequence alignment [18]. Experiments revealed that the knockout of this gene resulted in aborted seeds and, crucially, the generation of a proportion of maternal haploids in both hybrid and self-pollinated offspring. This confirmed that mutation of the *SlDMP* gene in tomato also confers an independent capacity for haploid induction. This approach was then successfully extended to allopolyploid species, with the establishment of DMP-based maternal haploid induction systems in Brassica napus and Nicotiana tabacum in 2022 [22]. Most recently, in 2024, a team from China Agricultural University achieved the first report of in vivo haploid induction in cucumber by editing the *CsDMP* gene [35]. However, it is noteworthy that the haploid induction efficiency achieved by editing DMP orthologs alone in dicots is generally lower than that obtained by knocking out MTL/ZmPLA1/NLD orthologs in monocots. However, the discovery of the *DMP* gene has opened up a completely new path for generating haploids in dicot plants through in vivo induction. In addition, haploid plants have been obtained from forest trees such as *Populus maximowiczii*, *Malus pumila*, and *Ziziphus jujuba* through anther culture in forest tree breeding [36,37,38]. With the development of in vitro culture and molecular cytology techniques, haploid breeding is becoming an important strategy to accelerate the crop breeding process. The application of haploid breeding in forest trees is relatively limited. However, obtaining homozygous diploid seeds through haploid breeding for propagation can solve problems caused by long-term asexual propagation, such as virus accumulation and cultivar degradation. For forest trees, propagating through pure-line seeds is also a good attempt in production.

The *DMP* gene is a gene that can regulate the development of plant germ cells, mutation or loss-of-function of this gene induces haploid production in plants. The essence of the *DMP* gene lies in its ability to regulate the plant meiosis process. Under normal circumstances, plants produce gametes with a diploid chromosome set through meiosis, whereas the mutation or loss of function of the *DMP* gene prevents the completion of meiosis, causing the plant to directly produce haploids. This mechanism provides an efficient technical means for plant breeding, especially in haploid breeding. Through haploid induction and chromosome doubling, homozygous breeding materials can be obtained in a relatively short period, significantly shortening the breeding cycle. To date, systematic analyses of this gene have been conducted in *Arachis hypogaea* [39] *Brassica oleracea* [40], *Nicotiana tabacum* [41], and *Brassica rapa* [42]. Furthermore, genome-wide expression analyses have been performed on it in various species, including *Beta vulgaris* [43], *Capsicum annuum* [44], *Ipomoea batatas* [45], *Cucumis sativus* [35], and *Oryza sativa* [46]. In zea, mutations in the *DMP* gene can significantly increase the haploid induction rate, thereby accelerating the breeding process. The application of haploid breeding technology has shortened the breeding cycle for maize varieties from multiple generations of selfing to only two generations, greatly improving breeding efficiency. Furthermore, the doubled progeny of haploid maize can rapidly fix desirable traits, facilitating the utilization of heterosis [20]. Potato is a tetraploid crop, and its traditional breeding is inefficient and genetic improvement is difficult. The introduction of the *DMP* gene enables the creation of highly pure diploid inbred lines in potato through haploid breeding technology, which opens up new possibilities for potato hybrid breeding. Through haploid induction, the potato breeding cycle is significantly shortened, and genomic homozygosity is increased, effectively solving the problems of self-incompatibility and inbreeding depression in traditional breeding [21]. In tomato, the application of the *DMP* gene has overcome the bottlenecks of small seeds and difficulty in haploid identification. The research team established a fluorescence-based screening technology that can accurately identify tomato haploids, thus accelerating the tomato breeding process [18]. Its discovery and application not only provide a new pathway for haploid breeding technology but also lay an important foundation for improving agricultural breeding efficiency.

In this article, a genome-wide analysis identified 11 *DMP* genes in the goji genome, which are unevenly distributed across seven chromosomes. To elucidate their evolutionary relationships, a phylogenetic tree was constructed using *DMP* proteins from goji and five other species: *Solanum lycopersicum*, *Arabidopsis*, *Cucumis saativus*, *Nicotiana tabacum*, and *Zea mays*. The analysis classified these proteins into three distinct subfamilies. Notably, the DMP family exhibited a clustering pattern specific to either monocots or dicots within each subfamily. This suggests that *DMP* genes shared a common ancestor prior to the divergence of monocotyledonous and dicotyledonous plants, a finding consistent with the results reported by Zhu et al. [16].

To comprehensively reveal the functional diversity of the LbDMP gene family and its potential role in enhancing seed vigor, we conducted a systematic, multi-dimensional analysis. The investigation began with the physicochemical properties of the proteins, revealing significant differences among LbDMP members. Combined with the diversification of their conserved motifs, this points to an evolutionary shift in the intrinsic properties of this gene family. Such evolutionary divergence suggests that different *LbDMP* genes may have evolved specialized biological roles in the growth and development of goji Subcellular localization predictions indicated that all *LbDMP* proteins are localized to the plasma membrane, which is consistent with the classic function of the DMP family as membrane proteins. Analysis of the *DMP* proteins’ secondary structures revealed that they are primarily composed of random coils and α-helices. Furthermore, the varying proportions of structural elements like α-helices and extended strands among the members lead to differences in their spatial folding patterns, providing a solid structural basis for their functional diversity. At the genomic level, 11 *LbDMP* genes are distributed unevenly across 7 chromosomes in a “telomere-enriched” pattern and are accompanied by tandem duplication events. This distribution pattern mirrors findings in oats, implying that gene amplification has played a driving role in the evolution of the DMP family [47]. Finally, analysis of cis-acting elements in the promoter regions revealed that these upstream regulatory elements act as “switches” for gene expression. They can respond to various environmental stresses and achieve tissue-specific regulation, thereby finely tuning the expression of *LbDMP* genes to adapt to complex internal and external environments.

In this study, LbDMP1 and LbDMP11 from goji were found to be closely related to AtDMP8, AtDMP9, and SlDMP8, clustering together on the same phylogenetic branch. This close relationship suggests that they may share conserved functions, potentially involving roles in plant pollination and fertilization, and the capacity to induce haploid formation. To further investigate their potential, qRT-PCR analysis was conducted to examine the expression patterns of LbDMP1 and LbDMP11 across various goji tissues. The results revealed that both genes were most highly expressed in pollen. This tissue-specific expression pattern further supports their identification as promising candidate genes for haploid induction in goji. Nevertheless, their functional role in haploid induction requires further validation.

The subcellular localization of a protein is often indicative of its biological function, making it a fundamental aspect of protein characterization. In *Arabidopsis thaliana*, the *DMP* proteins (DMP1, DMP2, DMP8, and DMP9) are primarily localized to membrane structures, including the endoplasmic reticulum (ER) and the tonoplast, suggesting that they function as secretory proteins [13]. To determine the subcellular localization of their homologs in goji, we constructed fusion expression vectors for LbDMP1 and LbDMP11 and transiently expressed them in tobacco leaves. Both prediction and experimental results showed that LbDMP1 localized to the plasma membrane, with a partial signal also detected in the endoplasmic reticulum. However, co-localization analysis revealed that its signal did not fully overlap with the ER marker. In contrast, LbDMP11 showed a clear and complete co-localization with the plasma membrane marker. Based on these findings, we speculate that both LbDMP1 and LbDMP11 likely function at the plasma membrane. This observation is consistent with the localization patterns reported for *DMP* proteins in *Arabidopsis*.

## 4. Materials and Methods

### 4.1. Identification of DMP Gene Family Members

The protein sequences of *DMP* genes from *Arabidopsis thaliana* (At), *Zea mays* (Zm), *Solanum lycopersicum* (Sl), *Nicotiana tabacum* (Nt), *Cucumis sativus* (Cs), and *Lycium barbarum* (Lb) were retrieved from several public databases. These included the *Arabidopsis* Information Resource (TAIR, https://www.arabidopsis.org), MaizeGDB (https://maizegdb.org), the Sol Genomics Network (SGN, https://solgenomics.net/), and the National Center for Biotechnology Information (NCBI, https://www.ncbi.nlm.nih.gov/). To identify *DMP* gene family members in goji, the known *Arabidopsis* DMP protein sequences were used as queries in a Blast search against the goji genome database. The resulting candidate sequences were then confirmed by screening for the presence of the conserved DUF679 domain using the Simple Modular Architecture Research Tool (SMART, https://smart.embl.de/, 21 September 2025) and the Conserved Domain Database (CDD, https://www.ncbi.nlm.nih.gov/Structure/cdd/wrpsb.cgi, 21 September 2025) [48]. Only sequences containing the complete DUF679 domain were considered as authentic *LbDMP* genes.

### 4.2. Analysis of Protein Physicochemical Properties and Subcellular Localization

The physicochemical properties of the identified DMP proteins were analyzed using the “Protein Parameter Calc” module within the Tbtools-II software suite. This analysis predicted key parameters, including molecular weight (MW), isoelectric point (pI), and instability index. For subcellular localization prediction, the Cell-PLoc 2.0 package (http://www.csbio.sjtu.edu.cn/bioinf/Cell-PLoc/, 21 September 2025) was employed to predict the potential cellular compartments of the LbDMP proteins.

### 4.3. Chromosomal Localization and Synteny Analysis of LbDMP Genes

The chromosomal locations of the *LbDMP* genes were determined from the goji genome annotation file. Subsequently, the Gene Density Profile tool within the TBtools-II software package was utilized to map the gene density across the *L. barbarum* chromosomes. The physical positions of the *LbDMP* genes on their respective chromosomes were visualized using the Gene Location Visualization tool, based on the provided GTF/GFF annotation files. To investigate the interspecies synteny (collinearity) between the *DMP* gene families of *L. barbarum* and *Zea mays*, the One Step MCScanX program, integrated into TBtools-II, was employed. The resulting synteny relationships were then visualized using the Multiple Synteny Plot function [49].

### 4.4. Analysis of Cis-Acting Elements

To identify potential regulatory elements, the 2000 bp genomic sequences upstream of the transcription start site of each goji *DMP* gene were extracted as putative promoter regions using the “Fasta Extract (Recommended)” module within TBtools-II. These sequences were then submitted to the online PlantCARE database (http://bioinformatics.psb.ugent.be/webtools/plantcare/html/, accessed on 21 September 2025) for the prediction of cis-acting elements [50]. The distribution and types of the identified cis-elements were subsequently visualized using TBtools-II. Furthermore, a dynamic, stacked heatmap was constructed using the BioLadder v2.0 online platform to illustrate the results.

### 4.5. Phylogenetic Analysis of the DMP Gene Family

Multiple sequence alignment of the DMP protein sequences was performed using the MUSCLE algorithm integrated within MEGA X software. Based on the resulting alignment, a phylogenetic tree was constructed by the Neighbor-Joining (NJ) method. The robustness of the tree topology was assessed with 1000 bootstrap replicates. The Poisson model was applied for distance calculation, and gaps/missing data were treated using the pairwise deletion option.

### 4.6. Multiple Sequence Alignment and Sequence Logo Generation

Multiple sequence alignment of the amino acid sequences from LbDMP1, LbDMP11, AtDMP8, AtDMP9, SlDMP8, NtDMP1, NtDMP2, and NtDMP3 was performed using MEGA software (version 6.06). Subsequently, the WebLogo 3 server (http://weblogo.threeplusone.com/, accessed on 21 September 2025) was utilized to generate a sequence logo, which visualizes the conservation of amino acids within the DUF679 domain.

### 4.7. Plant Material and Sampling

The plant material used in this study was goji, which was obtained from the Germplasm Repository of the Goji Research Institute, Ningxia Academy of Agriculture and Forestry Sciences, located at coordinates 38°64′ N, 106°15′ E. The plants were grown under standard field conditions. For the subsequent experiments, six distinct tissues—namely, leaves, petals, whole flowers, pedicels, pollen, and styles—were harvested from three-month-old *L. barbarum* ‘Ningqi-1’ plants at the onset of the flowering stage.

### 4.8. Subcellular Localization

For subcellular localization analysis, the recombinant plasmid containing the gene of interest was extracted from a verified monoclonal colony and introduced into *Agrobacterium* tumefaciens strain GV3101 via the freeze–thaw transformation method. A single positive transformant was selected and cultured overnight in 5 mL of LB liquid medium supplemented with rifampicin (25 μg/mL) and kanamycin (50 μg/mL). Bacterial cells were harvested by centrifugation and resuspended in an infiltration buffer (10 mM MgCl_2_, 10 mM MES, pH 5.7, and 200 µM acetosyringone (AS)). The cell suspension was adjusted to a final optical density at 600 nm (OD600) of 0.6 and incubated at room temperature for 3 h in the dark. The prepared *Agrobacterium* suspension was infiltrated into the abaxial epidermis of leaves from one-month-old *Nicotiana benthamiana* plants using a needleless syringe. The infiltrated plants were maintained in a dark chamber at 28 °C for 24 h, and then transferred to low-light conditions for another 24 h to allow for protein expression. The infiltrated leaf sections were then excised, mounted on microscope slides, and examined for fluorescence signals using a laser scanning confocal microscope. Fluorescence images were acquired using a Nikon laser scanning confocal microscope (Nikon C2-ER, Nikon Instruments Inc., Shanghai, China). The excitation/emission wavelengths were set to 488/510 nm for green fluorescence, 561/580 nm for mCherry and FMarker, and 640/675 nm for chlorophyll. Image processing was performed using Nikon NIS-Elements software Ver. 6.20.00 (Nikon Instruments Inc., Shanghai, China).

## 5. Conclusions

In this study, we identified a total of 11 *LbDMP* genes in the goji genome. These genes are unevenly distributed across seven chromosomes and can be classified into three distinct subfamilies based on phylogenetic analysis. Expression analysis via qRT-PCR revealed that LbDMP1 and LbDMP11 exhibit pollen-specific expression, marking them as promising candidate genes for future haploid breeding programs in goji. Subcellular localization experiments further demonstrated that LbDMP1 localizes to both the plasma membrane and the endoplasmic reticulum, whereas LbDMP11 is specifically targeted to the plasma membrane. While this study offers a preliminary characterization of the *DMP* gene family in goji, further functional validation is essential to fully elucidate their roles in various biological processes and to uncover the precise mechanisms by which they regulate development in this species. Despite the need for this future work, our findings lay a valuable theoretical foundation for advancing haploid breeding strategies in goji.

## Figures and Tables

**Table 1:** Subcellular localization prediction of *LbDMP* gene.

Gene Name	Gene ID	AA	Molecular Weight (KDa)	pI	Instability Index	Aliphatic Index	GRAVY	Predicted Sl Localization
LyDMP1	XM_060318353.1	227	24,957.34	8.68	35.54	88.77	0.294	Plasma membrane
LyDMP2	XM_060360897.1	225	24,612.39	9.32	34.93	93.24	0.093	Plasma membrane
LyDMP3	XM_060322142.1	214	24,424.93	4.86	51.05	96.12	0.243	Plasma membrane
LyDMP4	XM_060311640.1	237	26,143.01	7.64	44.85	95.86	0.079	Plasma membrane
LyDMP5	XM_060355464.1	223	24,259.37	6.99	44.55	102.83	0.29	Plasma membrane
LyDMP6	XM_060342180.1	232	25,832.99	6.37	38.84	102.11	0.112	Plasma membrane
LyDMP7	XM_060313040.1	178	19,204.23	8.62	29.47	88.65	0.343	Plasma membrane
LyDMP8	XM_060337220.1	210	22,994.79	5.14	37.18	102.62	0.407	Plasma membrane
LyDMP9	XM_060311640.1	237	26,143.01	7.64	44.85	95.86	0.079	Plasma membrane
LyDMP10	XM_059460021.1	234	26,398.39	9.09	34.11	95.26	0.392	Plasma membrane
LyDMP11	XM_060333010.1	227	24,892.22	8.52	35.7	89.21	0.311	Plasma membrane

## Data Availability

The datasets supporting the results presented in this manuscript are included within the article (and its Supplementary Materials).

## Supplementary Materials

The following supporting information can be downloaded at: https://www.mdpi.com/article/10.3390/ijms27062912/s1.
